# Editorial: Molecular mechanisms in breast cancer progression and metastasis

**DOI:** 10.3389/fonc.2022.991758

**Published:** 2022-08-05

**Authors:** Monica Fedele, Amy McCart Reed

**Affiliations:** ^1^ Institute for Experimental Endocrinology and Oncology (IEOS), National Research Council (CNR), Naples, Italy; ^2^ The University of Queensland, UQ Centre for Clinical Research, Brisbane, QL, Australia

**Keywords:** breast cancer, metastasis, epithelial- mesenchymal transition (EMT), lncRNA, tumour suppressor

Breast cancer progression and metastasis are the focus of intensive research, as only by increasing our understanding of the mechanisms and biology of these processes can we hope to appropriately intervene therapeutically. Both distant metastasis and progression are underpinned by the hallmarks of cancer (1, 2) and the papers contained in this Research Topic on Molecular Mechanisms in Breast Cancer Progression and Metastasis cover a number of these hallmarks, further pointing to the extensive complexity of breast cancer, as well as covering different stages of progression from early stage breast cancer to metastasis.

Long non-coding RNAs are emerging as cancer drivers across many cancer types and long-non-coding HOX transcript antisense intergenic RNA (HOTAIR) has been well studied in advanced cancers. It has been shown to bind to chromatin modifiers and repress transcription of target genes, as well as being a prognostic marker of lymph node metastases. The role of HOTAIR in the progression from *in situ* disease to frank carcinoma is just coming to light. Abba et al. present a detailed examination of the role of HOTAIR and demonstrate that its expression levels are upregulated when compared to matched normal. Indeed HOTAIR is one of the few genes to be differentially expressed between MCF10A and DCIS.COM (an *in situ* carcinoma model), and Abba et al. demonstrate using a raft of *in vitro* assays and comparative transcriptomics that it regulates bioprocesses including epithelial to mesenchymal transition (EMT) and migration, and is a positive regulator of cell growth and migration breast cells.

Centromere protein U (CENP-U) is an important cell-cycle regulator that participates in the assembly of the mature kinetochore. It has been studied in a range of cancers and Hao et al. provide data from a number of *in vitro* and *in vivo* analyses to show the oncogenic activity of CENP-U and its role in activating the PI3K/AKT/NFKB pathway.

Yang et al. describe ZNF213 (Zinc finger protein 213) as an important modulator of ERα signalling. Indeed, ZNF213 enhances the stability of ERα through the inhibition of its post-translational ubiquitinisation and may represent a future therapy target in ER positive breast cancers.

Taken together, studying across the gamut of cellular processes, including post-translational modifications, kinetochore assembly, and long non-coding RNAs, is furthering our understanding of breast cancer.

Another recently added hallmark of cancer that deals with tumor progression is metabolic reprogramming. In particular, cancer progression is closely linked to metabolism through the interplay between the EMT and metabolic reprogramming (3). Consistently, growing evidence associates metabolic diseases and tumor progression, including type 2 diabetes and an increased risk of breast cancer development and metastasis (4, 5), while the effects of some anti-hyperglycemic medications on risk of cancer development are still controversial (6).

In this frame, in the present Research Topic, two research papers by the same team explored the effect of two common antidiabetic dipeptidyl peptidase-4 inhibitors (DPP-4i), Saxgliptin (Sax) and Sitagliptin (Sit), on breast cancer progression. These compounds are currently recommended by the American Association of Clinical Endocrinology as first-line hypoglycemic drugs in type 2 diabetes mellitus. However, although their safety has been widely evaluated, long-term exposure to them can potentially exert unexpected effects on diabetic patients with cancer, including breast cancer. In the first study, using *in vitro* and *in vivo* breast cancer models, Li et al. demonstrate that Sax and Sit trigger oxidative stress through the overproduction of radical oxygen species (ROS). Overproduction of ROS induces abnormal NRF2 expression and activation, which enhances lung and liver metastasis under immunodeficient conditions. In the second study, Li et al. extended their exploration into immunocompetent mice, finding that antidiabetic DPP-4 inhibitors reprogram tumor microenvironment to enhance breast cancer metastasis *via* a ROS-NRF2-HO-1-NF-kB-NLRP3 axis. Activating this pathway also promotes tumor-infiltrating inflammation and immunosuppressive cells in metastatic sites, offering novel strategies to develop effective immunotherapy approaches to relieve DPP-4i-driven breast cancer metastasis.

Epithelial to mesenchymal transition (EMT) is an enduring hallmark of cancer, underpinning migration and invasion, and progression to a metastatic state. Li et al. and Liu et al. present *in vitro* studies examining the roles of key repressors of EMT. Extending previous work showing FOXA2’s role inhibiting metastasis, Liu et al. sought to identify FOXA2-interacting proteins using mass spectrometry. FOXP2 was identified, and then shown to also be an EMT repressor. FOXP2, interacting with FOXA2, activated expression of E-cadherin and restricted mesenchymal features.

The work of Li et al. demonstrates that the pseudokinase Nuclear Receptor Binding Protein 2 (NRBP2), is a breast cancer tumour suppressor, and its loss of expression is not in an indicator of a poor prognosis. NRBP2 was shown to impact cell proliferation, invasion and EMT in breast cancer cells. Overexpression of NRBP2 reduced lung metastases in nude mice, and its mechanism of action is likely through the AMPK/mTOR pathway, providing potential avenues of therapeutic intervention.

Overall, we believe that this collection opens new and interesting aspects of breast cancer progression and metastasis, and hope that the readers will enjoy it.

## Author contributions

MF and AR made an equal contribution to the writing and reviewing of this editorial. All authors contributed to the article and approved the submitted version.

## Acknowledgments

We thank the authors of the manuscripts and the expert Reviewers for their hard work.

## Conflict of interest

The authors declare that the research was conducted in the absence of any commercial or financial relationships that could be construed as a potential conflict of interest.

## Publisher’s note

All claims expressed in this article are solely those of the authors and do not necessarily represent those of their affiliated organizations, or those of the publisher, the editors and the reviewers. Any product that may be evaluated in this article, or claim that may be made by its manufacturer, is not guaranteed or endorsed by the publisher.
